# Healthcare avoidance during the early stages of the COVID-19 pandemic and all-cause mortality: a longitudinal community-based study

**DOI:** 10.3399/BJGP.2023.0637

**Published:** 2024-10-01

**Authors:** Marije J Splinter, Premysl Velek, Brenda CT Kieboom, M Arfan Ikram, Evelien IT de Schepper, M Kamran Ikram, Silvan Licher

**Affiliations:** Department of Epidemiology and Department of Neurology, Erasmus MC – University Medical Centre Rotterdam, Rotterdam, the Netherlands.; Department of Epidemiology and Department of General Practice, Erasmus MC – University Medical Centre Rotterdam, Rotterdam, the Netherlands.; Department of Epidemiology and Department of General Practice, Erasmus MC – University Medical Centre Rotterdam, Rotterdam, the Netherlands.; Department of Epidemiology, Erasmus MC – University Medical Centre Rotterdam, Rotterdam, the Netherlands.; Department of General Practice, Erasmus MC – University Medical Centre Rotterdam, Rotterdam, the Netherlands.; Department of Epidemiology and Department of Neurology, Erasmus MC – University Medical Centre Rotterdam, Rotterdam, the Netherlands.; Department of Epidemiology and Department of General Practice, Erasmus MC – University Medical Centre Rotterdam, Rotterdam, the Netherlands.

**Keywords:** Cohort studies, COVID-19, mortality, patient acceptance of health care, primary health care

## Abstract

**Background:**

During the COVID-19 pandemic, global trends of reduced healthcare-seeking behaviour were observed. This raises concerns about the consequences of healthcare avoidance for population health.

**Aim:**

To determine the association between healthcare avoidance during the early stages of the COVID-19 pandemic and all-cause mortality.

**Design and setting:**

This was a 32-month follow-up within the population-based Rotterdam Study, after sending a COVID-19 questionnaire at the onset of the pandemic in April 2020 to all communty dwelling participants (*n* = 6241/8732, response rate 71.5%).

**Method:**

Cox proportional hazards models assessed the risk of all-cause mortality among respondents who avoided health care because of the COVID-19 pandemic. Mortality status was collected through municipality registries and medical records.

**Results:**

Of 5656 respondents, one-fifth avoided health care because of the COVID-19 pandemic (*n* = 1143). Compared with non-avoiders, those who avoided health care more often reported symptoms of depression (*n* = 357, 31.2% versus *n* = 554, 12.3%) and anxiety (*n* = 340, 29.7% versus *n* = 549, 12.2%), and more often rated their health as poor to fair (*n* = 336, 29.4% versus *n* = 457, 10.1%) . Those who avoided health care had an increased adjusted risk of all-cause mortality (hazard ratio [HR] 1.30, 95% confidence interval [CI] = 1.01 to 1.67), which remained nearly identical after adjustment for history of any non-communicable disease (HR 1.20, 95% CI = 0.93 to 1.54). However, this association attenuated after additional adjustment for mental and physical self-perceived health factors (HR 0.93, 95% CI = 0.71 to 1.20).

**Conclusion:**

This study found an increased risk of all-cause mortality among individuals who avoided health care during COVID-19. These individuals were characterised by poor mental and physical self-perceived health. Therefore, interventions should be targeted to these vulnerable individuals to safeguard their access to primary and specialist care to limit health disparities, inside and beyond healthcare crises.

## Introduction

During the COVID-19 pandemic, global trends of reduced healthcare utilisation were observed.^1^^–^4 In a previous study among a large community-dwelling population, the authors of the current study showed that one in five individuals refrained from seeking medical attention during the COVID-19 pandemic, of whom one-third reported urgent symptoms indicative of stroke, heart disease, or cancer.^5^ Widespread concerns have been raised about the potential consequences of healthcare avoidance for population health, as unheeded symptoms could increase risks of adverse health outcomes.^6^ These concerns were demonstrated by the estimated 18.2 million excess deaths globally between 1 January 2020 and 31 December 2021, which is three times higher than the officially reported number of COVID-19-related deaths in that period.^7^ A part of this discrepancy might be explained by underreporting of COVID-19 deaths in certain countries. Significant increases in mortality from non-COVID-19-related causes also contributed to excess mortality, partially because of decreased admissions to hospital for routine care and deferral of preventive and elective health care.^8^ Furthermore, there is evidence for socioeconomic and mental health inequalities in mortality patterns during the COVID-19 pandemic.^9^^–^13 Yet, these observations do not fully explain the threefold difference in excess deaths.^7^

A potential missing piece to this puzzle is the contribution of patients’ healthcare-seeking behaviour,^1^ evaluated with reliable population-level data on healthcare avoidance, in relation to mortality. In this community-based cohort study, a 32-month follow-up of community-dwelling individuals who avoided health care during the early stages of the COVID-19 pandemic was conducted and whether this behaviour was related to increased risk of all-cause mortality was assessed.

**Table:** How this fits in

During the COVID-19 pandemic, trends of reduced healthcare-seeking behaviour were observed alongside global patterns of excess mortality, raising concerns about the consequences of healthcare avoidance for population health. This study found that individuals who avoided health care during COVID-19 were at an increased risk of all-cause mortality. Importantly, these individuals were characterised by underlying symptoms of depression and anxiety, as well as poor self-perceived health. The findings of this study emphasise the need for targeted interventions to safeguard access to primary and specialist care for these vulnerable individuals, during and beyond healthcare crises.

## Method

### Study population

This study was embedded in the ongoing population-based Rotterdam Study, a prospective cohort study aimed at investigating the aetiology and natural history of chronic diseases in mid-and late life.^14^ The Rotterdam Study was initiated in 1990 and consisted of 7983 residents of the district Ommoord in Rotterdam, the Netherlands, who were ≥55 years (RS-I). The cohort was expanded with additional study waves in 2000 (RS-II, *n* = 3011, ≥55 years), 2006 (RS-III, *n* = 3932, ≥45 years), and 2016 (RS-IV, *n* = 3005, ≥40 years).^14^ Since 1990, the total study population comprised 17 931 participants, all of whom were extensively examined at study entry and subsequent follow-up every 3–6 years.

This study is reported according to the Strengthening the Reporting of Observational Studies in Epidemiology (STROBE) guideline.

### Data collection

On 8 April 2020, all participants were identified who were still alive and actively involved in the Rotterdam Study (*n* = 9008). Of these, in the current study the authors included participants who were not admitted to hospital or living in a nursing home (*n* = 8732, 96.9%). On 20 April 2020 these participants were sent a dedicated COVID-19 questionnaire that covered subjects such as COVID-19-related symptoms and risk factors, lifestyle, mental health, and healthcare utilisation during the pandemic. A detailed description of the methods and validation of this questionnaire was published elsewhere.^15^

### Assessment of healthcare avoidance

Participants were asked whether they refrained from seeking medical attention from a GP or medical specialist in the weeks before filling out the questionnaire because of the COVID-19 pandemic, despite experiencing symptoms. The authors considered respondents (6241/8732, response rate 71.5%) who answered ‘yes’ to be ‘healthcare avoiders’ and the ‘no’ respondents formed the reference group. Additionally, healthcare utilisation of a subsample of participants who indicated that they avoided health care (889 out of 1143 participants) was verified by checking the medical records of the GP and medical specialists by hand. Detailed information about this verification process is available elsewhere.^5^

In the current study, the medical records of a subsample of the reference group were also reviewed to assess potential variation in healthcare utilisation, as this group could consist of participants who had symptoms but did not avoid health care; participants who did not experience any symptoms; or participants who avoided health care because of reasons unrelated to the COVID-19 pandemic. Three levels of certainty of non-avoidance were defined: ‘definite’, ‘probable’, and ‘possible’. Definite non-avoiders were participants who visited the GP or had a virtual consultation shortly after filling out the questionnaire, either in March or April 2020. Probable non-avoiders were participants who either had a virtual or physical consultation with the GP between May 2020 and March 2021. The remaining participants did not visit the GP from March 2020 to March 2021, and were labelled as possible non-avoiders.

### Assessment of all-cause mortality

For all the participants who filled in the question about healthcare avoidance (5656/6241, 90.6% of respondents), mortality status was collected until 1 January 2023. The date of death was obtained by notification from the municipal administration; GP; nursing home (in the cases of some participants, who moved into a nursing home after filling out the questionnaire); or from the family of the deceased. These follow-up data were complete through linkage with the Personal Records Database, a national population register maintained by municipalities that contains personal data for people who live in the Netherlands, including date of death. Living status was determined by the most recent date of a research visit, home interview, or magnetic resonance imaging scan for the Rotterdam Study; or the last date of checking the medical records of the GP by follow-up research assistants, whichever came first.

### Covariates

In addition to age and sex, all variables that the authors previously identified to be associated with healthcare avoidance as potential confounders were considered,^5^ including self-reported history of any non-communicable disease, educational attainment (primary education; low/intermediate general or lower vocational; intermediate vocational or higher general; higher vocational or university), occupational status (working; on sick leave; unemployed; retired; other), overall self-perceived health (poor; fair; good; very good; excellent), alcohol consumption and smoking status (self-reported use in the 2 weeks before filling out the questionnaire), symptoms of depression (using a shorter, validated version of the Centre for Epidemiological Studies Depression [CES-D] scale, the CES-D-10,^16^ weighted score ranging from 0 to 30), and symptoms of anxiety (using the validated anxiety subscale of the Hospital Anxiety and Depression Scale [HADS], the HADS-A^17^, weighted score ranging from 0 to 21).

### Statistical analyses

Characteristics of the study population that were measured on a continuous scale are reported as the mean and standard deviation (SD). Categorical variables are presented as the frequency and corresponding percentage of the total number of observations.

Whether individuals who avoided health care because of the COVID-19 pandemic had an increased risk of all-cause mortality was determined using multivariable Cox proportional hazards regression models expressed as hazard ratios (HR) with 95% confidence intervals (CIs). The proportional hazards assumption was assessed by examining the Schoenfeld residuals and plotting the log-minus-log survival curve.

The models were constructed in four different steps:

- model 1 was adjusted for age and sex;
- model 2 additionally for socioeconomic factors (occupational status and educational attainment);
- model 3 also for lifestyle factors (alcohol consumption and smoking) and self-reported history of any non-communicable disease; and
- model 4, additionally for overall self-perceived health and symptoms of depression and symptoms of anxiety.

Additionally, potential time-dependent variation in effect estimates was evaluated by censoring the follow-up time after 6, 12, 24, and 32 months. Missing values in covariates (all <3%) were imputed using the fully conditional specification method with a maximum of 10 iterations.

All statistical analyses were performed using SPSS version 28.0.1.0 and R version 4.2.2.

## Results

### Characteristics

Among 5656 participants who answered the question about healthcare avoidance, 1143 (20.2%) individuals reported that they had avoided seeking health care because of the COVID-19 pandemic despite experiencing symptoms for which they would have otherwise consulted a physician (Table 1). Compared with the reference group, these individuals were more often women (*n* = 758, 66.3% versus *n* = 2506, 55.5%), they more often considered their health to be poor to fair (336, 29.4% versus *n* = 457, 10.1%), and they more often experienced symptoms of depression (357, 31.2% versus *n* = 554, 12.3%) and anxiety (*n* = 340, 29.7% versus *n* = 549, 12.2%).

**Table 1.** Baseline characteristics of the study population

**Characteristic**	**All (*n* = 5656)**	**Avoidance group (*n* = 1143)**	**Reference group (*n* = 4513)**
**Age, years, mean (SD)**	69.4 (11.5)	71.7 (12.0)	68.8 (11.3)

**Women**	3264 (57.7)	758 (66.3)	2506 (55.5)

**Follow-up time in months, mean (SD)**	31.3 (4.0)	30.6 (5.4)	31.4 (3.6)

**Mortality status**			
Died	296 (5.2)	93 (8.1)	203 (4.5)
Alive on 1 January 2023	5360 (94.8)	1050 (91.9)	4310 (95.5)

**History of non-communicable diseases**			
Any	3659 (64.7)	889 (77.8)	2770 (61.4)
Cancer	812 (14.4)	211 (18.5)	601 (13.3)
Heart disease	1640 (29.0)	416 (36.4)	1224 (27.1)
Stroke	418 (7.4)	126 (11.0)	292 (6.5)
Chronic lung disease	795 (14.1)	226 (19.8)	569 (12.6)
Neurodegenerative disease	97 (1.7)	29 (2.5)	68 (1.5)
Diabetes	546 (9.7)	156 (13.6)	390 (8.6)
Mental illness	256 (4.5)	103 (9.0)	153 (3.4)
Other	1119 (19.8)	318 (27.8)	801 (17.7)

**Educational attainment**			
Primary education	343 (6.1)	102 (8.9)	241 (5.3)
Low/intermediate general or lower vocational	1874 (33.1)	421 (36.8)	1453 (32.2)
Intermediate vocational or higher general	1807 (31.9)	355 (31.1)	1452 (32.2)
Higher vocational or university	1579 (27.9)	249 (21.8)	1330 (29.5)
Missing	53 (0.9)	16 (1.4)	37 (0.8)

**Occupational status**			
Working (full time, part time, self-employed)	1580 (27.9)	204 (17.8)	1376 (30.5)
On sick leave	61 (1.1)	16 (1.4)	45 (1.0)
Unemployed	161 (2.8)	44 (3.8)	117 (2.6)
Retired	3406 (60.2)	723 (63.3)	2683 (59.5)
Other	289 (5.1)	92 (8.0)	197 (4.4)
Missing	159 (2.8)	64 (5.6)	95 (2.1)

**Overall self-perceived health**			
Poor	62 (1.1)	38 (3.3)	24 (0.5)
Fair	731 (12.9)	298 (26.1)	433 (9.6)
Good	3196 (56.5)	620 (54.2)	2576 (57.1)
Very good	1154 (20.4)	125 (10.9)	1029 (22.8)
Excellent	416 (7.4)	36 (3.1)	380 (8.4)
Missing	97 (1.7)	26 (2.3)	71 (1.6)

**Alcohol consumption within past 14 days**			
Yes	3094 (54.7)	548 (47.9)	2546 (56.4)
Missing	45 (0.8)	14 (1.2)	31 (0.7)

**Current smoking**			
Yes	592 (10.5)	135 (11.8)	457 (10.1)
Missing	39 (0.7)	10 (0.9)	29 (0.6)

**Symptoms of depression**			
Weighted score ≥10^a^	911 (16.1)	357 (31.2)	554 (12.3)
Missing	172 (3.0)	74 (6.5)	98 (2.2)

**Symptoms of anxiety**			
Weighted score ≥7^a^	889 (15.7)	340 (29.7)	549 (12.2)
Missing	115 (2.0)	47 (4.1)	68 (1.5)

*Data are n (%) unless otherwise specified.*

a *Validated cut-off scores at which individuals are considered to be at risk of clinical depression or anxiety. IQR = interquartile range. SD = standard deviation.*

Participants who did not fill in the question about healthcare avoidance (*n* = 585) were slightly older (72.7 versus 69.4 years), more often women (*n* = 379, 64.8% versus *n* = 3264, 57.7%), and had fewer years of educational attainment (*n* = 66, 11.3% versus *n* = 343, 6.1% primary education) (Supplementary Table S1).

Compared with responders, non-responders to the questionnaire (*n* = 2491) were younger (68.9 versus 70.2 years), more often women (*n* = 1521, 61.0% versus *n* = 3643, 58.0%), and had fewer years of educational attainment (*n* = 304, 12.2% versus *n* = 343, 6.1% primary education) (Supplementary Table S2).

Based on the review of 186 medical records of GPs from the reference group, it was found that only (*n* = 11) 5.9% of this reference group did not visit their GP or medical specialist in the year following the onset of the COVID-19 pandemic in the Netherlands (Table 2).

**Table 2.** Analysis of medical records of GPs from a subsample of the reference group (*n* = 186)

**Reference group**	***N* (%)**
**Possible non-avoidance**	11 (5.9)
**Probable non-avoidance**	93 (50.0)
**Definite non-avoidance**	82 (44.1)

### Healthcare avoidance and all-cause mortality

During a mean follow-up of 31.3 months (SD 4.0 months), 5.2% (*n* = 296/5656) of participants died. Among those who avoided health care because of the COVID-19 pandemic (*n* = 1143), 93 participants died (8.1%) (Table 1). Adjusted for age and sex, the healthcare avoiding group were at an increased risk of all-cause mortality (Table 3, model 1: HR 1.30, 95% CI = 1.01 to 1.67). Similar associations were observed when adjusting for educational attainment and occupational status (model 2: HR 1.29, 95% CI = 1.00 to 1.66). Adjustment for lifestyle factors and a history of any non-communicable disease did not materially change the estimates beyond loss of statistical significance (model 3: HR 1.20, 95% CI = 0.93 to 1.54).

**Table 3.** Healthcare avoidance and the risk of all-cause mortality in the general population (*n* = 5656)

**Statistical model**	**Follow-up, HR (95% CI)**			
	**6 months**	**12 months**	**24 months**	**32 months**
**Deaths, *n* (%)**	40 (0.7)	110 (1.9)	223 (3.9)	296 (5.2)
**Model 1 (adjusted for age and sex)**	2.19 (1.15 to 4.15)^a^	1.81 (1.22 to 2.68)^a^	1.42 (1.07 to 1.89)^a^	1.30 (1.01 to 1.67)^a^
**Model 2 (model 1, additionally adjusted for occupational status, educational attainment)**	2.02 (1.04 to 3.93)	1.86 (1.25 to 2.78)^a^	1.46 (1.09 to 1.96)^a^	1.29 (1.00 to 1.66)^a^
**Model 3 (model 2, additionally adjusted for alcohol consumption, smoking, and self-reported history of any non-communicable disease)**	1.68 (0.84 to 3.36)	1.72 (1.14 to 2.59)^a^	1.39 (1.03 to 1.87)^a^	1.20 (0.93 to 1.54)
**Model 4 (model 3, additionally adjusted for overall self-perceived health, symptoms of depression, symptoms of anxiety)**	1.07 (0.40 to 2.28)	1.14 (0.73 to 1.78)	0.95 (0.68 to 1.33)	0.93 (0.71 to 1.20)
**Model 4a (model 3, additionally adjusted for overall self-perceived health)**	1.24 (0.60 to 2.54)	1.35 (0.89 to 2.05)	1.09 (0.80 to 1.48)	1.00 (0.77 to 1.29)
**Model 4b (model 3, additionally adjusted for symptoms of depression)**	1.17 (0.56 to 2.46)	1.30 (0.84 to 2.01)	1.13 (0.82 to 1.55)	1.02 (0.79 to 1.32)
**Model 4c (model 3, additionally adjusted for symptoms of anxiety)**	1.45 (0.71 to 2.93)	1.46 (0.95 to 2.24)	1.27 (0.93 to 1.73)	1.19 (0.91 to 1.57)

a *P<0.05. HR = hazard ratio. CI = confidence interval. Self-perceived health = participants’ self-assessment of their overall health, which can encompass both mental and physical aspects*

However, additional adjustment for mental and self-perceived health factors weakened effect estimates (model 4: HR 0.93, 95% CI = 0.71 to 1.20). When these factors were assessed separately, associations attenuated in a model with correction for overall self-perceived health (model 4a: HR 1.00, 95% CI = 0.77 to 1.29) and symptoms of depression (model 4b: HR 1.02, 95% CI = 0.79 to 1.32), but not when adjusting for symptoms of anxiety (model 4c: HR 1.19, 95% CI = 0.91 to 1.57). Progressively extending the follow-up time from 6 to 32 months resulted in a slight decline of HR. However, given the overlap in confidence intervals across all four adjusted models, a time-dependent association between healthcare avoidance and all-cause mortality cannot be inferred.

## Discussion

### Summary

In this community-based study the risk of all-cause mortality was higher among individuals who avoided health care during the early stages of the COVID-19 pandemic compared with those who did not, adjusted for age, sex, occupational status, and educational attainment. Further adjustment for overall self-perceived health and symptoms of depression substantially attenuated this association. This implies that elevated rates of mortality were not directly attributable to healthcare avoidance, but rather stemmed from poor underlying mental and physical self-perceived health.

### Strengths and limitations

A major strength of this study is the direct assessment of self-reported healthcare-seeking behaviour and symptoms that were left unheeded through an extensive questionnaire, ensuring virtually complete follow-up of mortality status. Consequently, in this study the authors did not have to rely on indirect measures of healthcare utilisation by comparing registry data of consultation rates. A limitation is that healthcare avoidance and other behavioural covariates in the early stages of the pandemic were assessed, but the prevalence of these factors may have shifted over time, potentially affecting the results. A slight decline in HR on progressively extending the follow-up time was observed, however, given the overlap in confidence intervals the authors cannot state with certainty that the association between healthcare avoidance and all-cause mortality is time dependent. Importantly, existing literature indicates that depressive and anxiety disorders were more prevalent during lockdowns, but continued to occur at higher rates beyond those periods than would be expected based on pre-pandemic levels.^18^^–^21 Therefore, as those who avoided health care in this study more often reported having symptoms of depression and anxiety, the authors expect the direction of the associations to remain consistent. Further tracking of these individuals is required to fully understand the long-term implications of healthcare avoidance on population health.

Another limitation is limited generalisability of the findings to groups other than White, for example Black and minority ethnic groups, given that, 88.9% (*n* = 5027) of participants in this study were White. Future research should examine the relationship between healthcare-seeking behaviour and mortality in more diverse populations, considering that prior studies consistently have demonstrated higher rates of poor mental health and all-cause mortality among ethnic minority groups during the COVID-19 pandemic.^22^

### Comparison with existing literature

The authors of the current study previously demonstrated that symptoms of depression and self-perceived health were associated with healthcare avoidance during the COVID-19 pandemic.^5^ Other studies have confirmed that patients’ self-assessment of health status is a strong determinant of mortality among older adults, especially within 5 years of follow-up.^23^^–^25 Therefore, the current findings emphasise the potential added value of performing subjective health assessments alongside objective health measures for mortality.

In contrast, adjustment for history of any non-communicable disease did not meaningfully alter effect estimates except for loss of statistical significance. Even though individuals with chronic illnesses are generally at a higher risk of mortality, the association with healthcare-seeking behaviour may vary depending on the specific type of disease.^5^ On the one hand, certain patients have a higher susceptibility to a severe course of COVID-19, such as those with chronic lung diseases. Consequently, these patients may have been more inclined to avoid seeking medical attention out of fear of becoming infected. On the other hand, individuals with chronic diseases often exhibit higher rates of healthcare utilisation compared with those who do not have a chronic disease.^26^^,^^27^ For instance, individuals at risk of cardiovascular disease are frequently enrolled in cardiovascular risk management, which involves periodic check-ups conducted by their GP.^28^ Despite widespread cancellation or postponement of elective consultations during the COVID-19 pandemic, these patients may have higher awareness of urgent symptoms that require medical evaluation, distinguishing them from patients without such ongoing medical supervision.

### Implications for research and practice

During a 32-month follow-up period, an increased risk of all-cause mortality was observed among individuals who avoided health care during the early stages of the COVID-19 pandemic. Adjustment for mental and self-perceived health factors attenuated this association, implying that individuals who avoided health care were characterised by underlying vulnerability. Therefore, interventions aimed at mitigating the adverse consequences of healthcare avoidance should focus on improving access to primary and specialist care and on proactively targeting these vulnerable individuals to preserve their overall wellbeing and limit potential widening of health disparities.

## Data

Data from The Rotterdam Study can be made available to interested researchers upon request. Requests can be directed to the secretariat of the department of Epidemiology (secretariat.epi@erasmusmc.nl), or for more information visit http://www.ergo-onderzoek.nl/wp/contact. For legal and ethical restraints, and to ensure participant anonymity, we are unable to place data in a public repository.
